# Age, Operative Intent, and Mortality After Emergency Colorectal Cancer Surgery: An Exploratory Analysis of Age-Related Patterns

**DOI:** 10.3390/cancers18162646

**Published:** 2026-08-17

**Authors:** Vito Laterza, Marcello Covino, Carlo Alberto Schena, Davide Della Polla, Caterina Cina, Filomena Misuriello, Sergio Alfieri, Fausto Rosa

**Affiliations:** 1 Department of Digestive Surgery, Surgical Oncology and Liver Transplantation, University Hospital of Besançon, 25000 Besançon, France; vlaterza@chu-besancon.fr; 2 Department of Translational Medicine, Università Cattolica del Sacro Cuore, 00168 Rome, Italy; marcello.covino@unicatt.it (M.C.); sergioalfieri@unicatt.it (S.A.); 3 Department of Emergency Medicine, Fondazione Policlinico Universitario A. Gemelli IRCCS, 00168 Rome, Italy; davide.dellapolla@policlinicogemelli.it; 4 Department of General Surgery, Fondazione Poliambulanza Istituto Ospedaliero, 25123 Brescia, Italy; 5 Department of Surgery, Fondazione Policlinico Universitario A. Gemelli IRCCS, 00168 Rome, Italy; caterinacina@policlinicogemelli.it (C.C.); filomena.misuriello@guest.policlinicogemelli.it (F.M.)

**Keywords:** colorectal cancer, emergency surgery, mortality, age, palliative surgery

## Abstract

**Background:** Emergency surgery for complicated colorectal cancer (CRC) presenting with obstruction, perforation, or uncontrolled bleeding has high postoperative mortality, especially in older patients. However, age-related risk and the roles of radical versus palliative treatment are not fully understood. This study aimed to measure 90-day and 36-month mortality after emergency CRC surgery and to analyze whether the relationship between age and mortality is nonlinear and influenced by operative intent. **Methods:** Retrospective cohort of consecutive adults undergoing emergency surgery for complicated CRC (2015–2024). The outcome was 90-day and 36-month all-cause mortality. Cox regression provided adjusted hazard ratios (HR) using two models: a primary whole-cohort model omitting pathological T/N stage, and a secondary resection-only model including pathological stage. Age was modeled with restricted cubic splines and predicted 90-day and 36-month mortality were plotted by operative intent. **Results:** In 496 patients, 90-day mortality was 19.7% (98/496), while 36-month mortality was 37.3% (185/496). In the primary whole-cohort Cox model, independent predictors of 36-month mortality included age ≥75 years (HR 2.09, 95% CI 1.48–2.95, *p* < 0.001), Charlson Comorbidity Index (HR 1.02, 95% CI 0.98–1.06, *p* = 0.31), obstruction (HR 1.69, 95% CI 1.18–2.42, *p* = 0.004), perforation (HR 2.05, 95% CI 1.24–3.39, *p* = 0.005), and metastatic disease (HR 2.30, 95% CI 1.62–3.27, *p* < 0.001); radical operative intent was independently associated with lower 36-month mortality (HR 0.461, 95% CI 0.30–0.71, *p* = 0.001). In a secondary resection-only model additionally adjusting for pathological T/N stage (n = 426 with available pathology), the association for radical intent was attenuated and no longer statistically significant (HR 0.67, 95% CI 0.43–1.03, *p* = 0.067). The 36-month mortality spline estimate indicated an increasing risk with advancing age; a likelihood-ratio test did not show that the spline model fit significantly better than a linear age term (χ^2^ = 1.43, df = 1, *p* = 0.233), so this pattern is interpreted descriptively rather than as formal evidence of nonlinearity, and no formal interaction between age and operative intent was demonstrated. **Conclusions:** Ninety-day mortality after emergency colon cancer surgery remains significant. Age-related risk shows a predominantly monotonic, descriptive pattern and should not be assumed to vary by operative intent without formal interaction testing, emphasizing individualized risk assessment and shared decision-making.

## Simple Summary

Emergency surgery for complicated colorectal cancer carries high mortality. This study evaluated the impact of age and operative intent (radical vs. palliative) on 90-day and 36-month mortality in 496 patients. Overall, 90-day mortality was 19.7%; deaths within 90 days were more closely associated with acute physiological derangements and the severity of the initial presentation than with chronological age itself, although age remained an important determinant of outcomes over the longer term. However, 36-month survival was significantly impacted by advancing age, nodal stage, and metastatic disease. Using restricted cubic splines, we explored a predominantly monotonic relationship between age and mortality, with risk increasing more steeply in older patients (a likelihood-ratio test did not show that the spline model fit significantly better than a linear age term, χ^2^ = 1.43, df = 1, *p* = 0.233). Crucially, radical resection showed an independent association with lower 36-month mortality in the primary whole-cohort Cox model (HR 0.461, 95% CI 0.30–0.71, *p* = 0.001), chronological age alone did not uniformly preclude surgical benefit. These findings emphasize that age should not act as an absolute contraindication to curative-intent surgery. Instead, surgical decisions must be highly individualized, carefully balancing the patient’s acute physiological status, tumor burden, and baseline frailty.

## 1. Introduction

Colorectal cancer (CRC) remains a leading cause of cancer-related illness and death worldwide, especially in aging populations [1]. Many CRC cases present as emergencies, most often with large-bowel obstruction, and less frequently with perforation or uncontrolled bleeding [2]. Emergency presentation occurs in approximately 15–30% of cases [2,3] and is consistently linked to worse short- and long-term outcomes compared to elective surgery due to acute physiological stress, advanced disease stage, and limited time for preoperative optimization [4,5,6]. In older adults, aging, multiple health issues, and decreased organ reserve significantly affect postoperative tolerance and survival [7]. While elective CRC surgery in carefully selected elderly patients provides acceptable results, emergency surgery consistently shows much higher short-term mortality than elective procedures, with early postoperative deaths sometimes occurring beyond the typical 30-day period, such as with 90-day mortality reporting in colorectal surgery [8,9,10,11,12]. Studies indicate that emergency presentation, rather than age alone, is the main factor driving poor outcomes [13,14]. However, the relationship between age and surgical aggressiveness remains unclear, especially in emergencies where resection decisions must be made quickly and with incomplete staging information. This issue is particularly important for patients aged 75 or older, where the physical toll of surgery may outweigh its cancer-fighting benefits [15]. Current guidelines from the World Society of Emergency Surgery (WSES) and the American Society of Colon and Rectal Surgeons (ASCRS) recommend an individualized approach that considers tumor stage, overall health, and expected quality of life [3,16]. Yet, data on whether and how age influences the link between surgical intent (radical vs. non-radical) and early death is limited. Large population studies show that postoperative mortality increases with age [13,17,18]. Conversely, new evidence suggests that age-related frailty may act as a threshold rather than a continuous risk factor [19,20,21]. Rather than assuming a single age cutoff or a strictly non-linear age effect, formally testing the shape of the age–mortality relationship and its potential interaction with operative intent may offer better insights for emergency decisions and promote shared discussions about short-term risks.

This study aimed to assess short- and long-term surgical outcomes in patients undergoing emergency surgery for complicated CRC, examining the influence of age and surgical strategy on 90-day mortality and 36-month follow-up.

## 2. Materials and Methods

### 2.1. Study Design and Population

This is a single-center, retrospective observational study conducted at the Emergency Department (ED) of an urban teaching hospital. The hospital serves a catchment area of approximately 1.8 million residents and sees about 75,000 ED visits annually. Electronic medical records of all consecutive patients with CRC who underwent emergency surgery between January 2015 and December 2024 were retrospectively reviewed from a prospectively maintained database. Emergency indications included large bowel obstruction, perforation with diffuse peritonitis, perforation with localized abscess, and uncontrolled bleeding. All patients aged 18 years or older with CRC were included. Patients who arrived at the ED already intubated or in cardiocirculatory arrest were excluded. Data were handled in accordance with the Declaration of Helsinki. The study protocol was approved by the Institutional Review Board (Fondazione Policlinico Universitario Agostino Gemelli IRCCS, Rome, Italy, ID: 5121/2022), and informed consent was obtained in accordance with national regulations.

### 2.2. Data Collection and Study Variables

Demographic, clinical, operative, and pathological data were collected from electronic medical records, including age, sex, Charlson Comorbidity Index (CCI), ED presentation (obstruction, perforation, bleeding, abscess), operative details, postoperative course, and 90-day outcomes. Comorbidity burden was quantified using the original (unadjusted) Charlson Comorbidity Index, calculated from comorbidities documented in the electronic medical record at hospital admission. The age-adjustment component of the index was not applied, because age was analyzed separately as an independent covariate in all models. The National Early Warning Score (NEWS) was calculated from the first complete set of vital signs (respiratory rate, oxygen saturation, supplemental oxygen use, temperature, systolic blood pressure, heart rate, and level of consciousness) recorded at ED presentation, before any operative intervention. NEWS ≥ 4 was used as a binary indicator of acute physiological derangement at presentation. Bleeding was defined as gastrointestinal hemorrhage that could not be controlled with endoscopic hemostasis in hemodynamically unstable patients or those unresponsive to fluid resuscitation. Perforation with diffuse peritonitis was defined as the presence of free intraperitoneal air and fluid in all abdominal quadrants on CT imaging in patients who show signs of generalized peritonitis on physical examination. Perforation with abscess referred to patients with perforation and localized peritonitis or abscess who did not respond to conservative management with antibiotics. Obstruction was defined as a large-bowel blockage that could not be treated with endoscopy, such as colonic stent placement. Operative intent was categorized as either radical resection or palliative surgery. Palliative surgery was defined as exploratory laparotomy with or without enterostomy, bypass, or rescue resection. Formal radical resection was defined as tumor resection performed with curative intent together with an oncologically adequate lymphadenectomy. Rescue resection was defined as an emergency tumor resection performed primarily for source control without an oncologically adequate lymphadenectomy; because it did not meet the prespecified criteria for oncological radicality, rescue resection was classified within the palliative/non-radical group for the primary analysis. R0/R1/R2 resection status is a postoperative pathological outcome, distinct from the operative-intent variable, and is reported separately in Supplementary Table S4. Pathological staging (pTNM) was determined from the surgical specimen when a radical or rescue resection was performed. In procedures without tumor resection, pTNM was unavailable and recorded as missing. Clinical TNM staging based on preoperative CT was not used as the main variable because CT provides only moderate diagnostic accuracy for local staging, especially for nodal status in CRC [22,23]. Metastatic disease (M1) was defined as radiologically or intraoperatively documented distant metastatic disease. It was ascertained from preoperative contrast-enhanced CT of the chest, abdomen, and pelvis or, when imaging was inconclusive or unavailable given the emergency setting, from intraoperative findings (e.g., peritoneal carcinomatosis, liver metastases) recorded at the time of the index operation. Postoperative morbidity was evaluated within 30 days using the Clavien–Dindo classification; major complications were defined as Clavien–Dindo grade ≥ III. Clavien–Dindo grade V was considered perioperative or in-hospital death within 30 days and was thus distinguished from 90-day mortality. Vital status and date of death were confirmed by linking the hospital electronic medical records with the regional civil registry and death certificate database, which comprehensively records deaths both inside and outside the index institution. Ninety-day mortality was defined as all-cause death within 90 days of the index emergency operation. Overall survival was calculated from the date of the index emergency operation to death from any cause, or administrative censoring at 36 months. All patients entered the risk set at the time of surgery, including those who died within the first 90 days, thereby avoiding immortal-time bias that could arise from conditioning inclusion on hospital discharge or 90-day survival. This endpoint is hereafter referred to as 36-month overall survival/36-month all-cause mortality (follow-up censored at 36 months). Cause-specific mortality and recurrence were unavailable; therefore, cancer-specific survival and disease-free survival were not analyzed.

The following institutional context is provided to aid generalizability: endoscopic decompression and colonic stenting were available throughout the study period, and patients successfully managed with non-operative decompression were not included, since the study population comprised patients requiring emergency surgery. Formal geriatric or multidimensional frailty assessment was not systematically available throughout the study period; operative decisions were based on clinical assessment, physiological status, disease extent, technical resectability, and multidisciplinary/surgeon judgment. These center-specific pathways and the tertiary-referral setting may limit generalizability to centers with different endoscopic, geriatric, intensive-care, or surgical resources.

### 2.3. Study Endpoints

The primary endpoint was all-cause mortality, measured both at 90 days from the initial emergency operation and at 36 months. Secondary outcomes included the length of stay (LOS) for the index admission and admission to the intensive care unit (ICU).

### 2.4. Statistical Analysis

Continuous variables are presented as medians [interquartile range (IQR)] and compared using the Mann–Whitney U test. Categorical variables are shown as counts (percentages) and compared with the χ^2^ test or Fisher’s exact test, as appropriate. Time-to-event was calculated from the date of the initial emergency operation until death, with follow-up censored at 90 days for administrative reasons. Cox proportional hazards regression models were used to estimate hazard ratios (HR) with 95% confidence intervals (CI) for 90-day mortality. Two multivariable Cox models were used for 36-month mortality: (1) a primary whole-cohort model including age, CCI, obstruction, perforation, metastatic disease, and operative intent, applicable to all patients regardless of resection status; and (2) a secondary resection-only model additionally including pathological T and N stage, restricted to patients with available pathology. The designation of the whole-cohort model as the primary analysis was a post hoc decision, made after the complete-case (pathological-stage) model was found to systematically exclude the highest-risk non-resected palliative patients; it was not prespecified prior to data analysis. For each model we report the number of patients, number of deaths, and number excluded for missing data, and we compared baseline characteristics of included versus excluded patients (Supplementary Table S7). Proportional hazards assumptions were checked using Schoenfeld residuals. A likelihood-ratio test compared the fit of the restricted cubic spline age model against a model with age as a linear term, to formally assess evidence for nonlinearity. Median follow-up was estimated using the reverse Kaplan–Meier method (estimated median follow-up ≈ 24.6 months, IQR 9.8–34.1). We considered but did not perform propensity-score matching between radical and palliative groups, given unmeasured determinants of operative selection (frailty, contamination severity, hemodynamic instability, anticipated life expectancy) and limited common support between groups; findings from unadjusted and adjusted comparisons are therefore interpreted as associations subject to residual confounding by indication rather than causal treatment effects. A two-sided *p*-value less than 0.05 was deemed statistically significant.

### 2.5. Modeling the Age-Dependent Survival

To examine potential non-linear effects of age, we modeled age using restricted cubic splines within the Cox proportional hazards framework, by plotting the expected survival probabilities derived from the beta coefficients of multivariate Cox regression models. Adjusted 90-day probabilities of death were obtained from model-based survival estimates at t = 90 days (1–S(90|X)) and plotted by operative intent (radical resection versus palliative surgery). A restricted cubic spline (RCS) curve was fitted to describe this relationship, using age in years as a continuous predictor and adjusted mortality risk as the outcome. This non-parametric approach was selected to allow a flexible representation of the relationship without assuming a specific parametric form beforehand. Knots were placed at the 25th, 50th, and 75th percentiles of the age distribution, corresponding to ages of 62, 72, and 81 years (empirically derived from the age-decile distribution reported in Supplementary Table S5), with a reference age of 65 years. Spline curves are shown with 95% confidence bands and an accompanying age-distribution/rug plot (Supplementary Table S5) to flag regions of sparse data. Interaction between spline-transformed age and operative intent was not formally tested; stratified curves are therefore presented as exploratory and do not support inference about effect modification. Missing data were not imputed. An analogous method was used to estimate adjusted 36-month mortality probabilities.

### 2.6. Software

All statistical analyses were conducted with SPSS software v26^®^ (IBM, Armonk, NY, USA) and with custom Python 3.13 scripts (Python Software Foundation, Wilmington, DE, USA).

## 3. Results

A total of 496 patients were included in the study, of whom 360 (72.6%) underwent curative-intent surgery, and 136 (27.4%) received palliative care. Supplementary Table S1 compares the radical and palliative surgery groups. Obstruction was the most common complication in both groups, occurring in 43.1% and 53.7%, respectively. Among patients undergoing curative-intent surgery, right colectomy was the most frequent procedure at 50.6%, while palliative surgery was performed in 27.4% of the entire cohort. Regardless of surgical intent, a stoma was created in 42.7% of patients. The overall 90-day mortality rate was 19.7%, and the 36-month mortality rate was 37.3%.

Baseline characteristics and perioperative outcomes based on 90-day survival status are shown in Table 1. Of 496 patients, 98 had died by 90 days, and 398 had survived. Patients who died within 90 days more often presented with obstruction (53.1% vs. 48.0%, *p* = 0.01). Radical resection was performed less frequently in the deceased group (57.1% vs. 76.4%, *p* < 0.001), as was R0 resection (30.6% vs. 59.3%, *p* < 0.001). Conversely, stoma creation was more common in patients who died (60.2% vs. 38.4%, *p* < 0.001). Major postoperative complications (Clavien–Dindo ≥III) and ICU admission were more frequent among patients who died within 90 days (20.4% vs. 12.1%, *p* = 0.027; and 29.6% vs. 17.1%, *p* = 0.005, respectively), while LOS did not differ significantly.

Comparison between surviving and deceased patients at 36 months follow-up is detailed in Supplementary Table S2. At this follow-up, 185 patients had died, and 311 survived. Deceased patients more frequently presented with obstruction (51.9% vs. 42.4%, *p* = 0.04). Patients who survived tended to have lower tumor stages, received adjuvant chemotherapy, underwent resective surgery, achieved R0 resection, and had longer operative durations, all of which were significantly higher than those who died (*p* < 0.011, *p* = 0.011, *p* < 0.001, *p* < 0.001, *p* = 0.001, respectively). Conversely, stoma creation and major postoperative complications were significantly more common in deceased patients (*p* < 0.001 and *p* < 0.001, respectively).

Among patients who underwent radical resection (n = 360), 304 survived and 56 died within 90 days; among patients who underwent palliative surgery (n = 136), 94 survived and 42 died within 90 days (98 total 90-day deaths in the cohort of 496). A study flow diagram (Supplementary Figure S1) summarizes cohort composition, 90-day status, and 36-month status.

### 3.1. Descriptive Subgroup Analysis Among Patients Undergoing Radical Resection

The descriptive subgroup analysis (Supplementary Table S3) revealed that, among patients undergoing radical resection, age ≥ 75 years (69% vs. 34%, *p* < 0.001), higher comorbidity burden (median CCI: 9 vs. 7, *p* = 0.002), and obstructive presentation (59.3% vs. 35.6%, *p* < 0.001) were more common among those who died within 36 months than among survivors. Additionally, positive pathological nodal status (*p* < 0.001) and metastatic status (*p* < 0.001), along with non-R0 resections (43.4% vs. 18.2%, *p* < 0.001) and stoma creation (34.5% vs. 22.7%, *p* = 0.018), were more frequent among patients who died within 36 months. These comparisons are univariable and descriptive; no inferential claims of causality or model robustness are drawn from this subgroup analysis.

### 3.2. Cox Regression Analysis for Mortality at 36 Months

In the primary whole-cohort Cox model (Table 2, all 496 patients), radical operative intent was independently associated with lower 36-month mortality (HR 0.461, 95% CI 0.30–0.71, *p* = 0.001), after adjustment for age, CCI, obstruction, perforation, and metastatic disease (full estimates below). In a secondary resection-only model additionally including pathological T and N stage (n = 426 with available pathology; 70 patients [14.1%] excluded for missing pathological data), the association for radical intent was attenuated and no longer statistically significant (HR 0.67, 95% CI 0.43–1.03, *p* = 0.067), consistent with selection of the highest-risk, non-resected patients out of this complete-case model. Baseline comparison of included versus excluded patients is presented in Supplementary Table S7.

### 3.3. Analysis of Mortality

**Figure 1:**
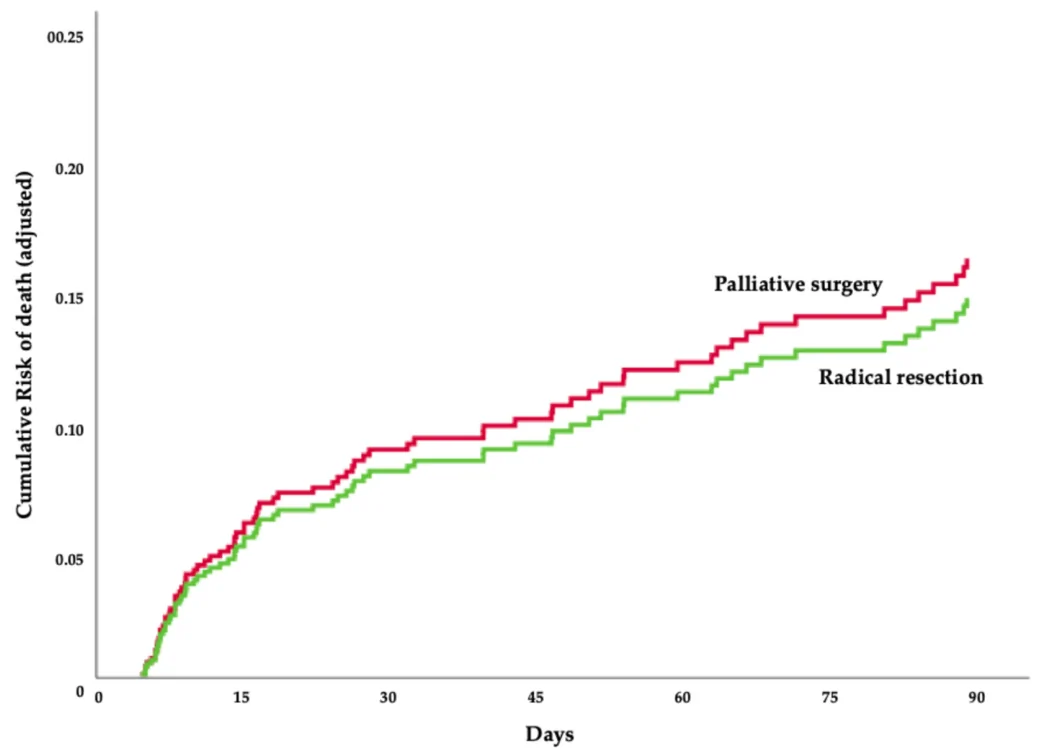
Model-based cumulative 90-day mortality after emergency surgery. The green curve represents patients undergoing radical surgery and the red curve those receiving palliative surgery. The difference between groups was not statistically significant (HR 0.91, 95% CI 0.43–1.91; *p* = 0.793).

**Figure 2:**
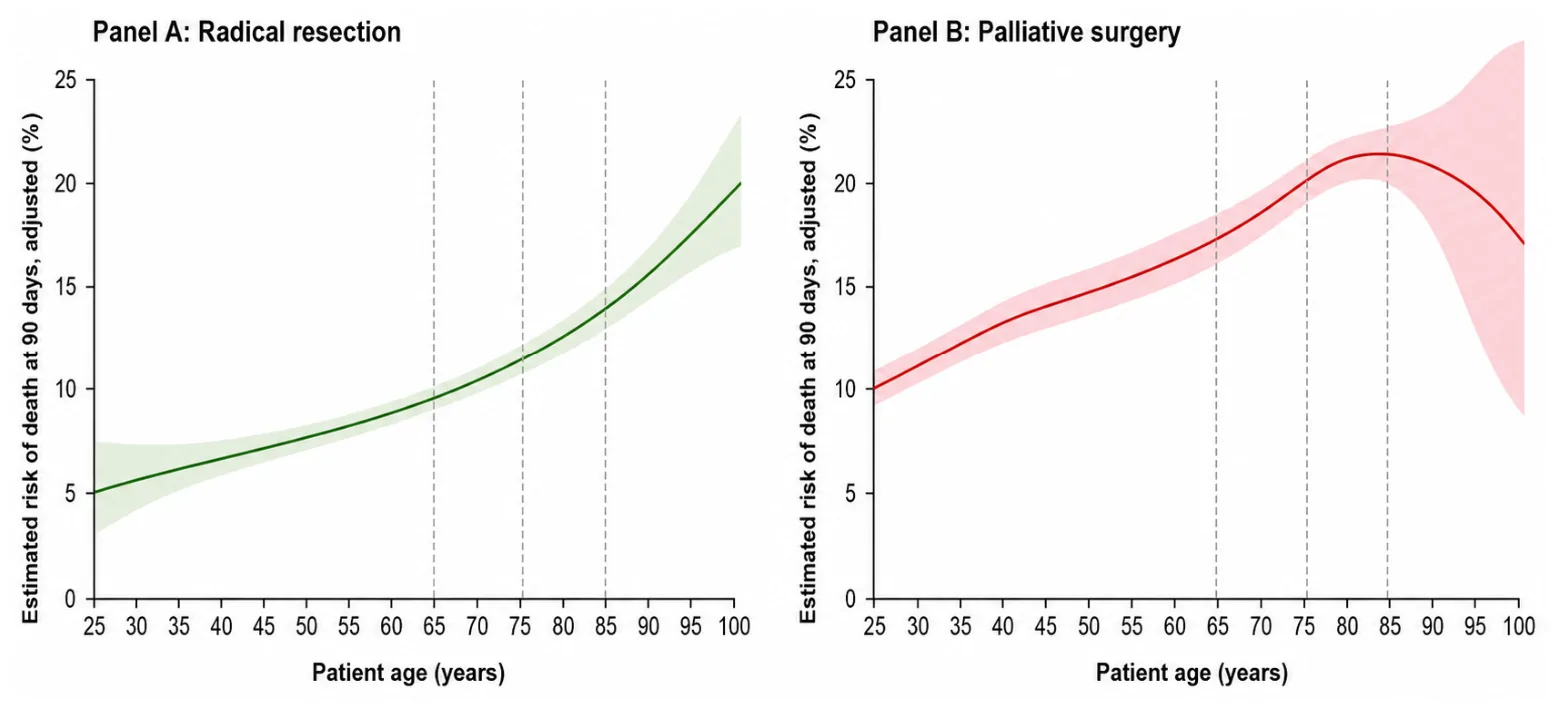
Model-based adjusted 90-day mortality risk according to patient age and operative intent. (**A**): radical resection (green curve); (**B**): palliative surgery (red curve). Curves are derived from fully adjusted models with reference age = 65 years; shaded bands show 95% confidence intervals; knot locations were 62, 72, and 81 years. Estimates at the extremes of the age distribution are based on sparse data (see Supplementary Table S5) and should be interpreted with caution. No formal interaction test was performed, and no inference of effect modification is intended.

**Figure 3:**
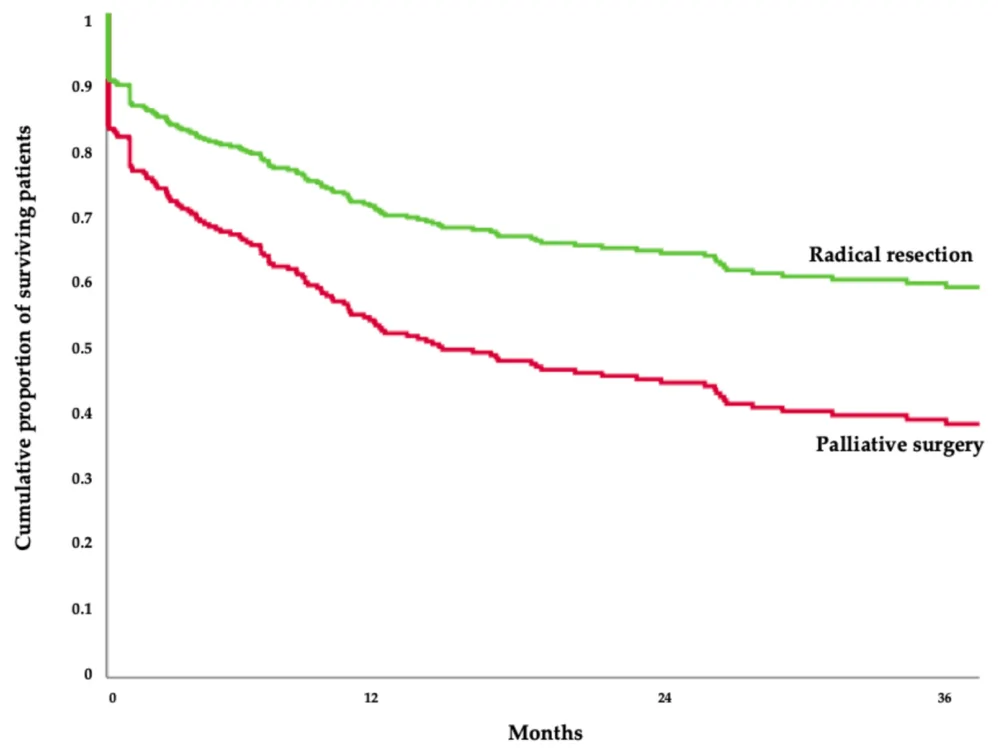
Cumulative 36-month survival according to operative intent. The green curve represents patients undergoing radical surgery, while the red curve indicates those undergoing palliative surgery. Patients undergoing radical resection showed higher cumulative survival than those receiving palliative surgery, with a significantly lower risk of death in this unadjusted Kaplan–Meier comparison (HR 0.54, 95% CI 0.37–0.78, *p* = 0.001); this estimate is descriptive and not adjusted for confounders.

**Figure 4:**
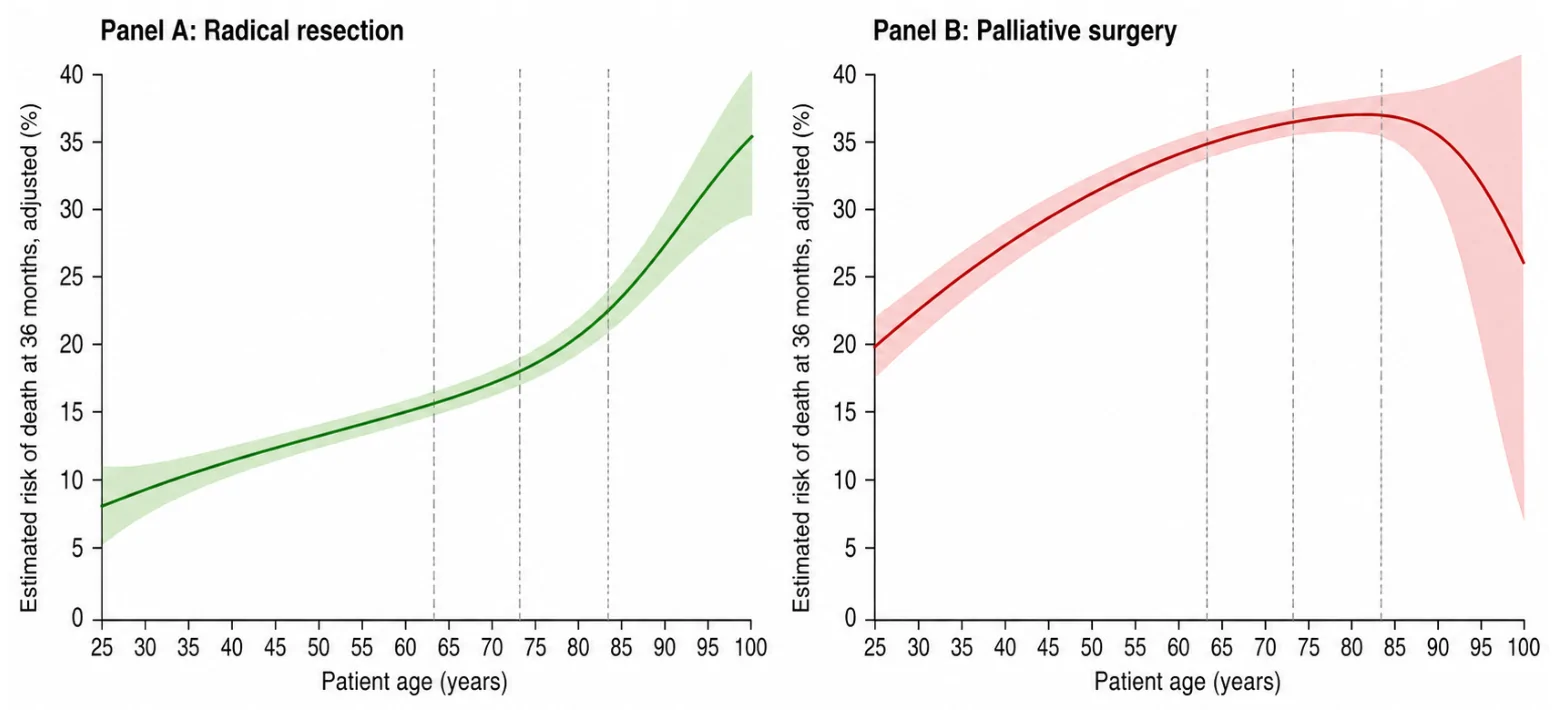
Age-dependent adjusted mortality risk by operative intent. (**A**): radical resection (green curve); (**B**): palliative surgery (red curve). Curves are derived from fully adjusted models with reference age = 65 years; shaded bands show 95% confidence intervals; knot locations were 62, 72, and 81 years. In the radical resection panel, estimated mortality risk increases progressively with age. In the palliative surgery panel, the curve shows wide confidence bands and an apparent decline at very advanced ages that most likely reflects sparse observations, survivor selection, and residual confounding by indication rather than a true protective effect (see Supplementary Table S5). No formal interaction test was performed, and no inference of effect modification is intended.

The adjusted 90-day mortality risk following surgical treatment is shown in Figure 1. Patients undergoing radical surgery had a numerically lower cumulative risk of death compared to those undergoing palliative surgery. However, this difference was not statistically significant (HR 0.91, 95% CI 0.43–1.91; *p* = 0.793). Figure 2 illustrates the relationship between patient age and the adjusted 90-day mortality risk by surgical approach. When examining 36-month survival (Figure 3), patients who underwent radical surgery demonstrated significantly improved cumulative survival compared with those who underwent palliative surgery. Radical surgery was associated with a significantly lower risk of death in this unadjusted Kaplan–Meier comparison (HR 0.54, 95% CI 0.37–0.78; *p* = 0.001; this estimate is descriptive and not adjusted for confounders). Figure 4 depicts the relationship between patient age and adjusted 36-month mortality risk by surgical approach.

## 4. Discussion

In this real-world cohort of patients undergoing emergency surgery for CRC, both 90-day and 36-month mortality were significant and remained clinically relevant beyond the early postoperative period. Mortality risk was strongly influenced by patient-level vulnerability (age), acute presentation (obstruction and perforation), disease burden (nodal/metastatic status), perioperative course (major postoperative complications), and flags of operative complexity (stoma creation). We considered 90-day mortality particularly informative in emergency oncologic surgery because it captures deaths occurring after discharge that may be missed by traditional 30-day metrics, aligning with recent literature advocating for extended follow-up to better estimate surgery-related risk [13,24]. The high early mortality seen in emergency CRC surgery aligns with population-based series showing significantly worse short-term outcomes after emergency compared to elective resection, reflecting both acute physiological stress and an adverse case mix, especially among older patients [25,26,27,28,29,30]. In our cohort, we observed a 19.7% 90-day mortality rate and a 37.3% 36-month mortality rate, consistent with previous emergency series [31,32].

Importantly, our results show that early postoperative mortality and 36-month survival are driven by partly different mechanisms. Specifically, unadjusted analysis paradoxically showed a younger age among patients who died within 90 days. This finding should not be interpreted as a protective effect of older age; it more likely reflects confounding by indication (more aggressive intervention in younger patients with severe acute presentations vs. more conservative management in frail elderly patients). These early deaths were characterized by a higher frequency of obstruction, lower rates of R0 resection, and higher surgical complexity (e.g., stoma creation, ICU admission). Therefore, younger patients dying early were likely disproportionately affected by severe acute physiological derangements or advanced initial oncologic burden rather than chronological age. Indeed, after adjustment for clinical and oncologic variables, no significant difference in 90-day mortality was observed between radical and palliative surgical approaches. These findings suggest that, in the immediate postoperative period, outcomes are strongly influenced by physiological stress and clinical instability, which may overshadow any incremental oncologic advantage of radical surgery in selected high-risk patients. We did not adjust the primary model for postoperative complications because these occur after the operative decision and may lie on the causal pathway between surgical strategy and mortality.

In contrast, 36-month survival differed significantly by operative intent and baseline vulnerability in the unadjusted comparison. The Kaplan–Meier analysis showed that patients undergoing radical resection had better overall survival than those receiving palliative surgery. In the primary whole-cohort Cox model, this survival advantage persisted as a statistically significant, independent association (HR 0.461, 95% CI 0.30–0.71, *p* = 0.001); it was only in the secondary resection-only model, which necessarily excludes the highest-risk non-resected palliative patients, that the association was attenuated to non-significance (HR 0.67, *p* = 0.067). This discrepancy illustrates how complete-case adjustment for pathological stage can introduce selection bias against the palliative group. Neither estimate should be regarded as definitive: the primary whole-cohort model may overestimate the benefit of radical resection because of residual confounding by indication (patients selected for radical surgery were healthier and had less advanced disease), while the secondary resection-only model may underestimate it because of differential exclusion of the highest-risk, non-resected palliative patients. The true association most plausibly lies between these two bounding estimates.

Modeling age flexibly with restricted cubic splines suggested a predominantly monotonic increase in risk with age; however, a likelihood-ratio test did not show that this spline model fit significantly better than a linear age term (χ^2^ = 1.43, df = 1, *p* = 0.233), so the pattern is best interpreted descriptively rather than as formal evidence of nonlinearity. At very advanced ages, the apparent convergence or partial reversal of the palliative-surgery curve should not be interpreted as evidence of a biologically lower mortality risk. Several non-mutually exclusive mechanisms may explain this pattern: strict selection bias whereby only older patients with comparatively favorable physiological status were offered surgery, and statistical instability of spline estimates at the distribution tails (see age-decile counts in Supplementary Table S5, where observations, particularly in the palliative group, become sparse above age 80).

Emergency presentations, such as obstruction or perforation, should be interpreted primarily as prognostic indicators rather than direct causes of mortality, as they often reflect illness severity and physiological disruption. Furthermore, the strong association between postoperative complications, stoma creation, and mortality is clinically intuitive and underscores the importance of early detection and post-discharge monitoring. Major complications, ICU admission, stoma creation, and operative duration are classified according to their temporal position relative to the operative decision (preoperative, intraoperative/treatment, or postoperative/mediator variables) and were not included in the primary model as baseline predictors, since adjustment for post-decision variables could block part of the causal pathway between operative strategy and mortality.

The clinical implication is that age should not be used as a sole contraindication to curative-intent surgery. In line with current emergency CRC guidelines [3,16], our findings support a structured individualized approach. In a clinically stable, physiologically fit patient with technically resectable disease, radical resection remains consistent with oncologic principles. Conversely, in very old or frail patients with severe sepsis, diffuse contamination, disseminated disease, poor functional reserve, or limited life expectancy, a staged, diverting, bypass, or other palliative/source-control strategy may better align operative burden with realistic prognostic expectations.

Of the 496 patients, 70 (14.1%) lacked pathological T/N stage and were excluded from the secondary resection-only Cox model; compared with included patients, excluded patients more frequently underwent palliative/non-radical procedures, had a higher prevalence of metastatic disease, and had slightly higher in-hospital and 90-day mortality (Supplementary Table S7), indicating that the resection-only model likely underestimates the true mortality risk associated with non-radical intent. We addressed this by making the whole-cohort model (excluding pathological stage) our primary analysis. We considered but did not perform propensity-score matching between radical and palliative groups because of unmeasured determinants of operative selection and limited common support; findings should be interpreted as associations subject to residual confounding by indication rather than causal treatment effects.

This study has limitations inherent to its retrospective observational design, including the risk of residual confounding by indication. Unmeasured factors such as baseline functional status, frailty, and detailed preoperative (e.g., lactate, vasopressor requirement, inflammatory markers) and intraoperative factors (e.g., degree of contamination, intraoperative hemodynamic instability) may have heavily influenced surgical decision-making and outcomes [33]. Moreover, formal frailty assessments, functional endpoints, and quality-of-life measures were unavailable, limiting our ability to evaluate patient-centered outcomes [19,20,34,35]. Finally, the analysis spanned 10 years, during which changes in clinical practice and perioperative care may have introduced temporal heterogeneity. This temporal heterogeneity may have influenced not only perioperative outcomes but also patient selection: evolving enhanced-recovery (ERAS) protocols and the progressive expansion of endoscopic colonic stenting over the study period could have shifted both the age and comorbidity profile of patients selected for emergency surgery and the observed mortality trends over time. A formal calendar-period adjustment was beyond the scope of this analysis and represents an area for future research. Despite these limitations, the large real-world cohort, the pre-specified definitions of surgical indications, and the flexible modeling of age using restricted cubic splines enhance the clinical relevance of our findings and lessen dependence on arbitrary age thresholds.

## 5. Conclusions

In conclusion, emergency surgery for CRC carries a high risk of mortality. Modeling age with restricted cubic splines suggested a predominantly monotonic, descriptive increase in 36-month mortality risk with age, without formal statistical evidence of nonlinearity or of effect modification by operative intent. These findings highlight the importance of structured perioperative risk assessment and targeted strategies to prevent major complications, which could help lower mortality rates in the highest-risk groups.

## Figures and Tables

**Table 1:** Comparison between surviving and deceased patients within 90 days of the index surgical operation.

	Alive (N = 398)	Deceased (N = 98)	*p* Value
**Age (years)**	71 (60–79)	65 (57–80)	**0.004**
**Age ≥ 75**	170 (42.7)	36 (36.7)	0.29
**Sex (Male)**	213 (53.5)	59 (60.2)	0.14
**CCI**	7 (5–10)	6 (5–10)	0.68
**Diagnosis at ED admission**			
Bleeding	76 (19.1)	22 (22.4)	0.09
Perforation with diffuse peritonitis	81 (20.4)	18 (18.4)	0.37
Perforation with abscess	50 (12.6)	6 (6.1)	0.2
Obstruction	191 (48.0)	52 (53.1)	**0.01**
**Staging**			
pT			0.07 °
1	3 (0.7)	0 (0)	
2	7 (1.7)	0 (0)	
3	154 (38.7)	31 (31.6)	
4	184 (46.2)	40 (40.8)	
pN			**<0.001** *
0	89 (22.4)	3 (3.1)	
1	90 (22.6)	12 (12.2)	
2	121 (30.4)	34 (34.7)	
pM			**0.013** ^§^
0	266 (66.8)	49 (50)	
1	82 (20.6)	22 (22.4)	
**Chemotherapy**			
Neoadjuvant	42 (10.6)	10 (10.2)	0.92
Adjuvant	189 (47.5)	30 (30.6)	**0.002**
**Type of Surgery**			
Radical Surgery	304 (76.4)	56 (57.1)	**<0.001**
Palliative Surgery ^@^	94 (23.6)	42 (42.9)	
**Radicality**			
Radicality R 0	236 (59.3)	30 (30.6)	
Radicality R 1	14 (3.5)	6 (6.1)	**<0.001**
Radicality R 2	148 (37.2)	62 (63.3)	
**Stoma creation**	153 (38.4)	59 (60.2)	**<0.001**
**Surgery Duration (min.)**	150 (99–210)	120 (92–174)	**0.006**
**Surgical Complications**			
Clavien Dindo 0–2	350 (87.9)	78 (79.6)	**0.027**
Clavien Dindo ≥ 3	48 (12.1)	20 (20.4)	
**Outcomes**			
ICU admission	68 (17.1)	29 (29.6)	**0.005**
LOS (days)	11.5 (7.5–18.5)	11 (7.3–19)	0.99

Abbreviations: CCI, Charlson Comorbidity Index; ED, Emergency Department; LOS, length of stay; ICU, Intensive Care Unit; Note: Values are expressed as median (IQR) for continuous variables and counts (%) for categorical variables. ^@^ Exploratory Laparotomy w/o enterostomy w/o by-pass w/o rescue resection. ° pT1-2 vs. pT3-4 * pN0 vs. pN1-2 ^§^ pM0 vs. pM1.

**Table 2:** Primary whole-cohort Cox model for 36-month mortality.

	All Patients	
Variable	HR (95% CI)	*p* Value
Age (≥75)	2.09 (1.48–2.95)	**<0.001**
CCI	1.02 (0.98–1.06)	0.31
Perforation	2.05 (1.24–3.39)	**0.005**
Obstruction	1.69 (1.18–2.42)	**0.004**
Metastatic disease	2.30 (1.62–3.27)	**<0.001**
Radical surgery	0.461 (0.30–0.71)	**0.** **001**

Abbreviations: CCI, Charlson Comorbidity Index; HR, Hazard Ratio. The primary model (all patients) omits pathological T/N stage, which was unavailable for non-resected patients.

## Data Availability

The data presented in this study are available on request from the corresponding authors due to legal reasons.

## Supplementary Materials

The following supporting information can be downloaded at https://www.mdpi.com/article/10.3390/cancers18162646/s1, Table S1: Comparison between radical and palliative surgery; Table S2: Comparison between surviving versus deceased patients at 36 months; Table S3: Comparison between living and deceased patients undergoing radical surgery at 36 months; Table S4: Classification of operative strategy and resection status (R0/R1/R2/non-resective) for the whole cohort; Table S5: Number of patients and deaths by age decile, overall and by operative intent; Table S6: Full multivariable Cox model for 90-day mortality (n = 496 patients; 98 events [deaths within 90 days]); Table S7: Comparison of patients included vs. excluded from the secondary pathological-stage Cox model; Figure S1: Study flow diagram.

## Abbreviations

The following abbreviations are used in this manuscript:

**CRC**	Colorectal Cancer
**HR**	Hazard Ratio
**CI**	Confidence Interval
**WSES**	World Society of Emergency Surgery
**ASCRS**	American Society of Colon and Rectal Surgeons
**ED**	Emergency Department
**CCI**	Charlson Comorbidity Index
**pTNM**	Pathological Tumor, Node, Metastasis
**LOS**	Length of Stay
**ICU**	Intensive Care Unit
**IQR**	Interquartile Range
**SPSS**	Statistical Package for the Social Sciences
