# Supplementary material for: Age, Operative Intent, and Mortality After Emergency Colorectal Cancer Surgery: An Exploratory Analysis of Age-Related Patterns
Source: Cancers (Basel). 2026 Aug 17;18(16):2646. doi: 10.3390/cancers18162646 (PMC13510549; doi:10.3390/cancers18162646)
Supplement: Supplementary file 1 [file cancers-18-02646-s001.zip › cancers-4485690-supplementary.pdf]

Supplementary Materials: Age, Operative Intent, and Mortality After Emergency Colorectal Cancer Surgery: An Exploratory Analysis of Age-Related Patterns

Vito Laterza [1,†], Marcello Covino [2,3,†], Carlo Alberto Schena [4,*], Davide Della Polla [3], Caterina Cina [5], Filomena Misuriello [5], Sergio Alfieri [2,5] and Fausto Rosa [2,5,*]

Table S1. Comparison between radical and palliative surgery.

	Palliative[@] (N=136)	Radical (N= 360)
**Age (years)**	68 [61–76]	73 [62–81]
**Age ≥75 years**	37 (27.2)	161 (44.7)
**Sex (Male)**	85 (62.5)	187 (51.9)
**CCI**	7 [5–9]	7 [4–10]
**Diagnosis at the Emergency Department admission**		
Bleeding	16 (11.8)	68 (18.9)
Perforation with diffuse peritonitis	26 (19.1)	103 (28.6)
Perforation with abscess	21 (15.4)	34 (9.4)
Obstruction	73 (53.7)	155 (43.1)
**Staging (pTNM)**		
pT		
1	0 (0)	5 (1.4)
2	0 (0)	9 (2.5)
3	6 (4.4)	206 (57.2)
4	53 (39.0)	140 (38.9)
pN		
0	0 (0)	141 (39.2)
1	13 (9.6)	147 (40.8)
2	46 (33.8)	72 (20)
pM		
0	19 (14.0)	286 (79.4)
1	40 (29.4)	74 (20.6)
**Chemotherapy**		
Neoadjuvant	40 (29.4)	12 (3.3)
Adjuvant	55 (40.4)	164 (45.6)
**Type of Surgery**		
Palliative/Non-resected[@]	136 (100)	0 (0)
Right colectomy	0 (0)	182 (50.6)
Left colectomy	0 (0)	65 (18.1)
Anterior Rectal Resection	0 (0)	97 (26.9)
Total Colectomy	0 (0)	16 (4.4)
Stoma creation	117 (86)	95 (26.4)
Surgery Duration (min.)	77.5 [52–109]	170 [130–222]
**Surgical Complications**	24* (17.6)	108* (30)
Clavien–Dindo 0	47 (34.6)	102 (28.3)
Clavien–Dindo 1	56 (41.2)	127 (35.3)
Clavien–Dindo 2	16 (11.8)	80 (22.2)
Clavien–Dindo 3	7 (5.1)	19 (5.3)
Clavien–Dindo 4	1 (0.7)	24 (6.7)
Clavien–Dindo 5	9 (6.6)	8 (2.2)
**Outcomes**		

Death (90-day)	42 (30.9)	56 (15.5)
Death (36-month)	72 (52.9)	113 (31.4)
LOS (days)	10.1 [5.6–18.4]	11.4 [8–18.9]
ICU admission	14 (10.3)	83 (23.1)
Time survival (months)	2.8 [0.8–17]	9.7 [1.4–39.9]
Follow up (months)	2.6 [0–16.4]	9.74 [1.4–39.9]

Table S1 is intended to be descriptive. Because groups are defined by outcome and/or treatment selection (not randomized), hypothesis testing of baseline differences may be misleading; therefore, p-values are not reported in Table 1.

Abbreviations: CCI, Charlson Comorbidity Index; LOS, length of stay; ICU, Intensive Care Unit. Note: Values are expressed as median [IQR] for continuous variables and counts (%) for categorical variables.@ Exploratory Laparotomy w/o enterostomy w/o by-pass w/o rescue resection. * The number of patients shown for total complications is less than the sum of the patients listed as having individual complications, because many patients had more than one complication.

Table S2. Comparison between surviving versus deceased patients at 36 months.

	**Alive (N=311)**	**Deceased (N=185)**	***P value***
**Age (years)**	69 [58–77]	71 [57–80]	0.616
**Age ≥75 years**	116 (37.3)	82 (44.3)	0.147
**Sex (male)**	163 (52.4)	109 (58.9)	0.159
**CCI**	7.00 [4.00–10.00]	8 [5.00–10.00]	0.1
**Diagnosis at the Emergency Department admission**			
Bleeding	56 (18)	28 (15.1)	0.41
Perforation with diffuse peritonitis	86 (27.7)	43 (23.2)	0.26
Perforation with abscess	37 (11.9)	18 (9.7)	0.46
Obstruction	132 (42.4)	96 (51.9)	**0.04**
**Staging**			
pT			0.065
1	3 (1)	0 (0)	
2	7 (2.3)	0 (0)	
3	103 (33.1)	82 (44.3)	
4	138 (44.4)	86 (46.5)	
pN			**<0.001**
0	79 (25.4)	13 (7.0)	
1	63 (20.3)	39 (21.1)	
2	107 (34.4)	48 (25.9)	
pM			**<0.001**
0	246 (79.1)	69 (37.3)	
1	42 (13.5)	62 (33.5)	
**Chemotherapy**			
Neoadjuvant	29 (9.3)	23 (12.4)	0.275
Adjuvant	151 (48.6)	68 (36.8)	**0.011**
**Type of surgery**			**<0.001**
Resective Surgery	247 (79.4)	113 (61.1)	
Palliative Surgery@	64 (20.6)	72 (38.9)	

Radicality R0	202 (64.9)	64 (34.6)	**<0.001**
Radicality R1	24 (7.7)	7 (3.8)	
Radicality R2	85 (27.3)	114 (61.6)	
**Stoma creation**	107 (34.4)	105 (56.8)	**<0.001**
**Surgery duration (min)**	156 [105–210]	127.5 [85.5–194.25]	**0.001**
**Surgical complications**			
Clavien–Dindo 0-2	274 (88.1)	154 (83.2)	**<0.001**
Clavien–Dindo ≥ 3	37 (11.9)	31 (16.8)	
**Outcomes**			
ICU admission	53 (17.0)	44 (23.8)	0.067
LOS (days)	11.1 [7.55–18.00]	12.10 [7.40–20.20]	0.461

Abbreviations: CCI, Charlson Comorbidity Index; LOS, length of stay; ICU, Intensive Care Unit. Note: Values expressed as median (IQR) for continuous variables and counts (%) for categorical variables. @ Exploratory Laparotomy w/o enterostomy w/o by-pass w/o rescue resection.

Table S3. Comparison between living and deceased patients undergoing radical surgery at 36 months.

	**Alive (N=247)**	**Deceased (N=113)**	***P* value**
**Age**	71.00 [61.00–80.00]	75.00 [65.00–83.00]	0.057
**Age ≥75 years**	84 (34)	78 (69)	**<0.001**
**Sex (Male)**	123 (49.8)	64 (56.6)	0.228
**CCI**	7.00 [4.00–10.00]	9.00 [6.00–11.00]	**0.002**
**Diagnosis at the Emergency Department admission**			
Bleeding	53 (21.5)	15 (13.3)	0.066
Perforation with diffuse peritonitis	78 (31.6)	25 (22.1)	0.065
Perforation with abscess	28 (11.3)	6 (5.3)	0.070
Obstruction	88 (35.6)	67 (59.3)	**<0.001**
**Staging (pTNM)**			
pT			0.553°
1	4 (1.6)	1 (0.9)	
2	8 (3.2)	1 (0.9)	
3	140 (56.7)	66 (58.4)	
4	95 (38.5)	45 (39.8)	
pN			**<0.001***
0	91 (44.4)	17 (19.1)	
1	71 (34.6)	43 (48.3)	
2	43 (21.0)	29 (32.6)	
pM			**<0.001**§
0	210 (85)	76 (67.3)	
1	37 (15)	37 (32.7)	
**Chemotherapy**			
Neoadjuvant	7 (2.8)	5 (4.4)	0.435
Adjuvant	120 (48.6)	44 (38.9)	0.088
**Radical Surgery (Intention to treat)**			**<0.001**

Radicality R 0	202 (81.8)	64 (56.6)	
Radicality R 1-2	45 (18.2)	49 (43.4)	
**Type of Surgery**			0.098
Right colectomy	127 (51.4)	55 (48.7)	
Left colectomy	44 (17.8)	21 (18.6)	
Anterior Rectal Resection	70 (28.3)	27 (23.9)	
Total Colectomy	6 (2.5)	10 (8.8)	
Stoma creation	56 (22.7)	39 (34.5)	**0.018**
Surgery Duration (min.)	172.00 [133.00–224.00]	159.00 [120.00–215.00]	0.353
**Surgical Complications**	64 (25.9)	44 (38.9)	**0.012**
Clavien–Dindo 0	78 (31.6)	24 (21.2)	**<0.001**[#]
Clavien–Dindo 1	90 (36.4)	37 (32.7)	
Clavien–Dindo 2	45 (18.2)	35 (31)	
Clavien–Dindo 3	14 (5.7)	5 (4.4)	
Clavien–Dindo 4	20 (8.1)	4 (3.5)	
Clavien–Dindo 5	0 (0)	8 (7.1)	
**Outcomes**			
LOS (days)	11.32 [8.00–18.15]	12.44 [8.00–20.44]	0.325
ICU admission	51 (20.6)	32 (28.3)	0.109

Abbreviations: CCI, Charlson Comorbidity Index; LOS, length of stay; ICU, Intensive Care Unit.

Note: Values expressed as median [IQR] for continuous variables and n (%) for categorical variables.

[*] The number of patients shown for total complications is less than the sum of the patients listed as having individual complications, because many patients had more than one complication.

[#] Clavien–Dindo 0-2 vs 3-5.

°pT1-2 vs pT3-4.

*pN0 vs pN1-2.

§ pM0 vs pM1.

# Clavien–Dindo 0-2 vs 3-5.

Table S4. Classification of operative strategy and resection status (R0/R1/R2/non-resective) for the whole cohort.

Category	n	%
R0 resection	266	53.6
R1 resection	20	4.0
R2 resection / rescue resection (gross residual disease)	60	12.1
Non-resective procedure (exploratory laparotomy, diversion, or bypass)	150	30.2
Total	496	100.0

Table S5. Number of patients and deaths by age decile, overall and by operative intent.

Age decile	Total n	Radical n	Palliative n	Deaths (36-mo) total	Deaths radical	Deaths palliative
20–29	3	1	2	2	1	1
30–39	8	4	4	7	3	4
40–49	21	12	9	9	4	5
50–59	68	45	23	13	7	6
60–69	122	93	29	46	30	16
70–79	141	111	30	45	31	14
80–89	107	78	29	49	30	19
90–99	26	16	10	14	7	7
Total	496	360	136	185	113	72

Table S6. Full multivariable Cox model for 90-day mortality (n = 496 patients; 98 events [deaths within 90 days]).

Variable	HR	95% CI	P value
Age	1.017	1.004–1.030	0.008
Charlson Comorbidity Index	1.045	0.997–1.095	0.064
NEWS ≥4	1.099	0.813–1.485	0.539
Perforation	1.733	1.136–2.644	0.011
Metastatic disease	1.722	1.239–2.393	0.001
Palliative surgery	2.421	1.766–3.319	<0.001

All covariates were fully ascertained for the entire cohort (n=496) from structured electronic medical records; no missing data or exclusions applied to this model.

Table S7. Comparison of patients included vs. excluded from the secondary pathological-stage Cox model.

Characteristic	Included (n=426)	Excluded (n=70)
Total, n (%)	426 (85.9)	70 (14.1)
Reason for exclusion: non-resective procedure (exploratory laparotomy, diversion, or bypass, no tumor resection), n	—	66
Reason for exclusion: other missing pathological data, n	—	4
Operative intent — Radical resection, n (%)	356 (83.6)	4 (5.7)
Operative intent — Palliative surgery, n (%)	70 (16.4)	66 (94.3)
Age (years), median [IQR]	71 [61–80]	66 [55–78]
Metastatic disease (pM1 or clinical/intraoperative), n (%)	78 (18.3)	26 (37.1)
In-hospital (30-day) mortality, n (%)	34 (8.0)	9 (12.9)
90-day mortality, n (%)	78 (18.3)	20 (28.6)
36-month mortality, n (%)	149 (35.0)	36 (51.4)

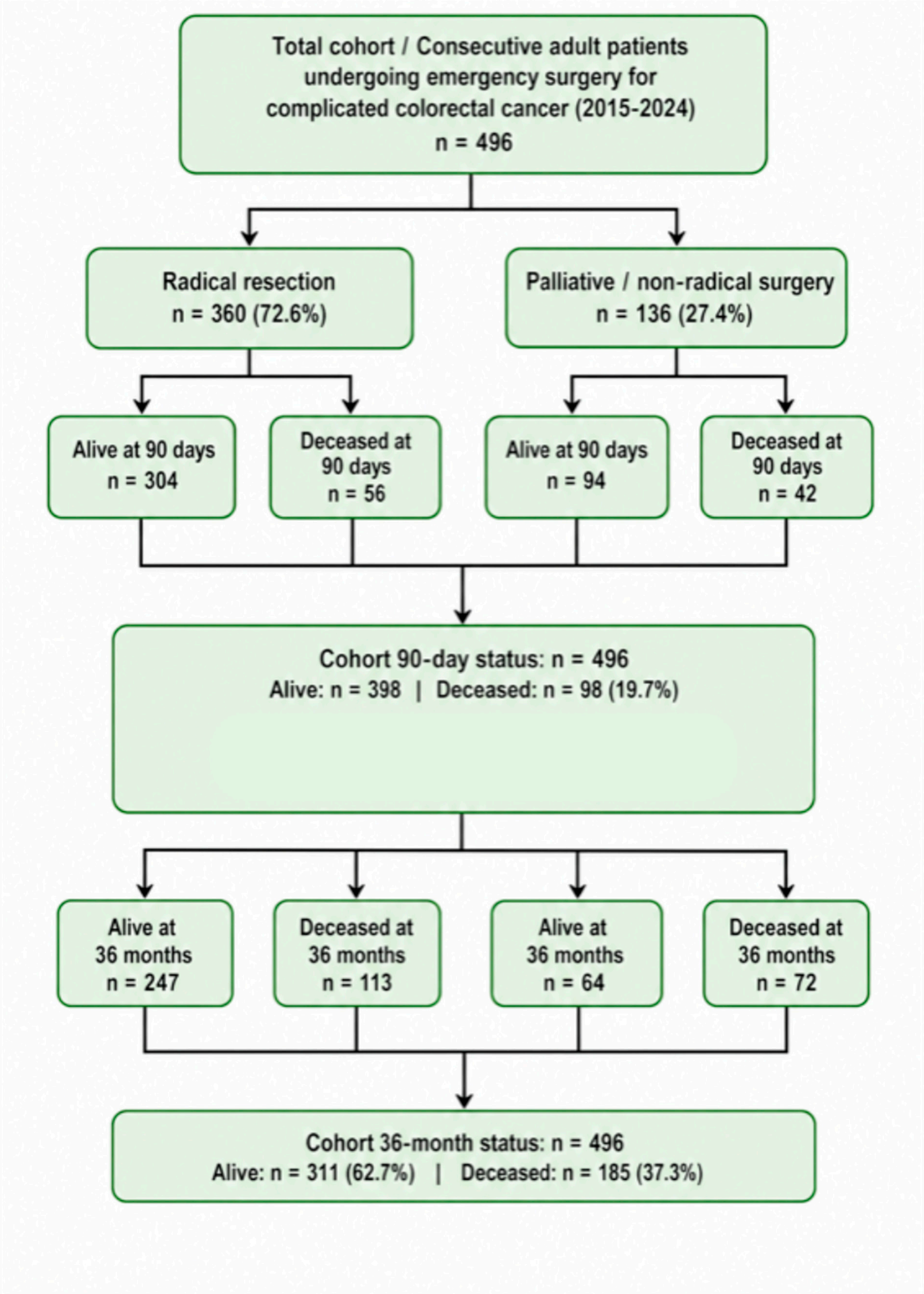

Figure S1. Study flow diagram.
